# Mr. Guy, on Ascites

**Published:** 1808-09

**Authors:** William Guy

**Affiliations:** Chichester


### To the Editors of the Medical and Physical Journal.

GENTLEMEN,

SHOULD a vacant page occur in your valuable Journal, I will thank you to employ it in submitting the following remarks, respecting the Treatment of Ascites, for the consideration of our medical Brethren. And, should the plan recommended be adopted, and found to succeed with them, as it has with me, I shall sit down with the pleasing reflection, that I have contributed, in some small degree at least, towards the alleviation of human misery.

I am, &c.

WILLIAM GUY.

*Chichester, August* 14, 1804.

---

It is, I believe, generally admitted, that Paracentesis is very frequently merely a palliative remedy; that the relief it affords is but of short duration; and that when performed, by the most expert and able practitioners, it has occasionally even proved fatal; still, however, the operation in many cases cannot be dispensed with. My only object, therefore, is to prevent a needless repetition of it, by shewing, that the water may be daily evacuated, with perfect ease and safety to the patient, by the following simple method. As soon as the fluid has been discharged by the operation, I introduce through the canula a bougie, upon which, having withdrawn the former, I introduce another canula rendered air tight, by means of a small plug inserted at the outer end, over which a cap is made to fit exactly. In order to retain it in its proper situation, a circular piece of adhesive plaster, spread on leather, about five inches in diameter, (the lower part of which may, for obvious reasons, require to be lessened), is fixed to the canula by suture, holes for that purpose being made in the circular plate. A belt, nine inches wide, made of horse's girth, with straps and buckles, should be applied previous to the operation, and gradually tightened as the water runs off; which is the the only bandage required. It should have in it a circular hole as large as a crown piece, to admit the top of the canula.

This mode of conducting the business I adopted above a twelvemonth ago, and I am free to confess, that I then considered myself in full possession of the claim to originality; but on further inquiry I found, that Celsus had been before hand with me; for he, "having made a wound into the abdomen with an iron instrument, introduced a leaden leaden or brazen pipe, through which, the water was to be evacuated; and he directs, that when the greater part of it was discharged, the pipe should be stopped with a bit of linen, and left in the wound; then, on the following days, a hemina (about three-quarters of a pint) is to be let out every day, till no water appears to remain."

After this I cannot profess to have made a discovery; and should any merit attach, it can only be derived from the revival of a practice, to which very few trials appear to have been given, and which has long since been discarded; but, as far as I am capable of judging, without its advantages having been duly appreciated.

The chief objection to this plan has been, the fear of putrefaction taking place in the contained fluids, from the admission of air into the abdomen. But surely the same objection must obtrude itself with full force, against the very frequent repetition of paracentesis in the same patient; an example of which is recorded in your Journal for the year 1803. The operation of drawing off the water is the same in both cases; and as the canula I recommend is air tight, and closed as soon as a full stream even begins to lessen, I do not hesitate to assert, that, with these precautions, the above objection is perfectly obviated. In fact, I have put it to the test in several cases, where the water has been regularly drawn off every, or every other, day, according to its accumulation, even for a month together, which took place in an old woman of seventy, and she obtained a permanent cure.

Before I close this paper, I will briefly hint at one or two material advantages, which may probably result from the mode of treatment I have taken the liberty to recommend.

Will it not prove beneficial, although the cause of the ascites may be unsurmountable, by mitigating considerably those severe symptoms depending solely on excessive distension?

Does it not give room for remedies to operate? And will it not afford a better opportunity to the medical practitioner, of steadily pursuing those means he may deem best adapted to specific cases; instead of having his views counteracted by the necesssary, but too often, unavailing endeavours, to lessen the daily accumulation of a fluid, which in the course of a few minutes may be so easily evacuted?

I may probably, on a future day, take the liberty of troubling you with a few more remarks on the subject; at present my sole aim is, to submit the plan of treatment to

 the the Faculty, who, I am persuaded, will give it a fair trial; and to request that through the medium of your interesting Journal, the public may be informed of the result.

---

The canula may be made of silver, or ivory; in size, it should be exactly the same with that part of the trocar, which is enclosed in its proper canula, and which is to be used in the operation. In length, from the circular plate surrounding it, one inch and a quarter, which part enters the abdomen. The part external to the plate, over which a cap is screwed, or nicely fitted, is half an inch; but I think this part projecting from the body, small as it is, may admit of being still a little shortened; the advantage of which is obvious. Although that part of the canula which penetrates the cavity is open, still three or four small holes, as in a catheter, will be beneficial.

